# Enhanced MERS Coronavirus Surveillance of Travelers from the Middle East to England

**DOI:** 10.3201/eid2009.140817

**Published:** 2014-09

**Authors:** Helen Lucy Thomas, Hongxin Zhao, Helen K. Green, Nicola L. Boddington, Carlos F.A. Carvalho, Husam K. Osman, Carol Sadler, Maria Zambon, Alison Bermingham, Richard G. Pebody

**Affiliations:** Public Health England, London, UK (H.L. Thomas, H. Zhao, H.K. Green, N.L. Boddington, C.F.A. Carvalho, C. Sadler, M. Zambon, A. Bermingham, and R.G. Pebody);; Public Health England, Birmingham, UK (H.K. Osman)

**Keywords:** Public health surveillance, Middle East respiratory syndrome, MERS, coronavirus, Middle East, England, United Kingdom, viruses

## Abstract

During the first year of enhanced MERS coronavirus surveillance in England, 77 persons traveling from the Middle East had acute respiratory illness and were tested for the virus. Infection was confirmed in 2 travelers with acute respiratory distress syndrome and 2 of their contacts. Patients with less severe manifestations tested negative.

The first reported case-patient infected with Middle East respiratory syndrome coronavirus (MERS-CoV) died in June 2012 in the Kingdom of Saudi Arabia and was reported on September 20 (*1*). The second case reported globally was in a Qatari national patient who had been transferred from Qatar to a hospital in England; preliminary data sharing on September 23 indicated that isolates from the second case-patient had 99.5% identity with the virus identified in the first case. (*2*). On September 24, 2012, Public Health England (PHE) (formerly the Health Protection Agency [HPA]) established an enhanced surveillance system to rapidly detect and investigate possible cases of MERS-CoV infection among travelers to England from the Middle East. The first 12 months of surveillance in England identified 1 additional case of MERS-CoV in a traveler returning from the Middle East and 2 cases among family contacts of this second case.

Definitions for possible and confirmed cases were established. Possible cases were defined by clinical and epidemiologic criteria. Clinical criteria specified acute respiratory syndrome (including fever ≥38°C or history of fever and cough) requiring hospitalization and clinical or radiologic evidence prompting suspicion of lower airway involvement not explained by another etiology. Epidemiologic criteria specified travel to or residence in an area where infection with MERS-CoV could have been acquired during the 10 days before onset of illness. At the time these criteria were initiated, the Kingdom of Saudi Arabia and Qatar were the 2 areas indicated. A confirmed case was defined by respiratory samples testing positive for MERS-CoV by at least 2 specific PCR assays targeting different regions of the MERS-CoV genome.

Because MERS-CoV is an emerging pathogen, case definitions were, and continue to be, revised in response to new information (*2*), in agreement with World Health Organization case definitions (*3*–*5*). Substantial revisions included extension of the geographic areas where infection could have occurred to include all countries neighboring those where infection could have been acquired (November 29, 2012), the recommendation to test patients with the appropriate clinical and epidemiologic criteria if they had an alternative etiology which did not fully explain their clinical manifestation (February 12, 2013), and extension of the incubation period to 14 days (June 21, 2013). 

## The Study

Enhanced surveillance involved the collection of a minimum dataset for each possible case, including demographic data, clinical symptoms, travel and contact history, and results of testing for respiratory pathogens (*6*). Nose and throat swab specimens and, when possible, lower respiratory tract specimens, were tested at 1 of 4 regional laboratories. Although the testing guidelines recommended MERS-CoV testing after exclusion of alternative etiologies, other tests were conducted in parallel with MERS-CoV testing for most suspected cases.

During the first few days of surveillance, a pan-coronavirus assay conducted at the PHE National Reference Laboratory was used as a screening test; then the viral genome was fully sequenced. After the generation of MERS-CoV specific assays, a first-line screening assay targeting the viral genomic area upstream of the E gene (*7*) was conducted, followed by confirmatory testing at the HPA/PHE National Reference Laboratory. Results of MERS-CoV testing are reported regularly in the HPA/PHE Weekly Influenza Report (*8*).

A descriptive analysis included the number of persons tested and proportion positive for MERS-CoV by key demographic, epidemiologic, and clinical characteristics. The positive predictive value of different combinations of signs and symptoms was calculated as the proportion of persons who had signs and symptoms of MERS and tested positive for MERS-CoV and showed exact (Clopper-Pearson) binomial 95% confidence intervals.

During September 24, 2012–October 15, 2013, 77 travelers from the Middle East that met the possible case definition were tested for MERS-CoV. Seventy-five travelers tested negative on the screening assay, and 2 tested positive. Positive results on the screening assay were confirmed by positive results at the HPA/PHE National Reference Laboratory. 

In addition to testing the 77 persons who met all of the possible case criteria, MERS-CoV testing was conducted on 13 patients who had severe acute respiratory disease but did not meet the travel requirements: 2 had a travel history outside the Middle East, 4 had no travel history in the relevant exposure period, and travel histories of the remaining 7 were unknown. MERS-CoV was not detected in any of these persons.

The clinical and epidemiologic characteristics of the 77 persons tested and their MERS-CoV test results are shown in Table 1. Those tested ranged in age from 3 months to 90 years; 34 (44%) had signs of pulmonary parenchymal involvement. The 2 confirmed cases were in male patients, 45 and 60 years of age; both had severe acute respiratory symptoms requiring treatment by extracorporeal membrane oxygenation; both subsequently died. MERS-CoV PCR testing was conducted on 53 contacts of the 2 confirmed case-patients in England; 2 of these contacts tested MERS-CoV positive (*9*,*10*).

**Table 1:** Results of MERS coronavirus testing of 77 travelers from the Middle East to England by key clinical and epidemiologic characteristics where information available, September 2012–October 2013

Characteristics	No. tested	No. (%) MERS coronavirus–positive
Age group, y		
0–4	5	0
5–17	1	0
18–44	10	0
45–64	34	2 (6)
≥65	25	0
Unknown	2	0
Sex		
M	49	2 (4)
F	26	0
Unknown	2	0
Clinical history		
Fever	51	2 (4)
Cough	51	2 (4)
Pulmonary parenchymal involvement	34	2 (6)
Acute respiratory distress syndrome	7	2 (29)
Mechanical ventilation	15	2 (13)
Extracorporeal membrane oxygenation	2	2 (100)
		
Travel history in exposure period* before symptom onset†		
Israel	1	0
Jordan	1	0
Kingdom of Saudi Arabia	40	1 (3)
Qatar	7	1 (14)
United Arab Emirates	27	0
Yemen	1	0

*Exposure period was 10 days until June 2013, when it was increased to 14 days. †Some patients had also traveled to countries outside the Middle East.

The positive predictive value for MERS-CoV infection of different combinations of signs and symptoms is shown in Table 2. No case-patients who did not have pulmonary parenchymal involvement tested positive for MERS-CoV, and the positive predictive value of the clinical manifestations increased as the severity of disease increased. Of the 77 patients tested, 22 had positive results for alternative respiratory pathogens, including 10 with influenza (7 influenza A and 3 influenza B); 1 of the influenza A–infected case-patients was later confirmed to also be infected with MERS-CoV. Two case-patients tested positive for *Legionella pneumophila*, 4 for rhinovirus, 3 for adenovirus, 1 for respiratory syncytial virus, and 1 for human metapneumovirus.

**Table 2:** Positive predictive value of signs and symptoms among 77 travelers from the Middle East tested for MERS-CoV, Enhanced MERS-CoV Surveillance System, England, September 2012–October 2013*

Signs and symptoms	No. MERS CoV–positive/no. tested	Positive predictive value, % (95% CI)
Fever, cough; no pulmonary parenchymal involvement	0/3	0 (0–71†)
Fever, cough, and pulmonary parenchymal involvement	2/18	11 (1–35)
Fever, cough, and pulmonary parenchymal involvement requiring mechanical ventilation	2/4	50 (7–93)
Fever, cough, pulmonary parenchymal involvement, and acute respiratory distress syndrome	2/4	50 (7–93)
Fever, cough, and pulmonary parenchymal involvement requiring extracorporeal membrane oxygenation	2/2	100 (16–100)


*MERS-CoV, Middle East respiratory syndrome coronavirus. †1-sided, 97.5% CI.

## Conclusions

Unlike surveillance for established organisms, surveillance for a novel pathogen requires analysis of information collected from all patients tested, even from those that test negative, to build knowledge of the predictive value of different epidemiologic and clinical manifestations. This report on the characteristics of patients traveling to England from the Middle East and tested for MERS-CoV enables a first crude estimation of the positive predictive value of different signs and symptoms during the first year following the emergence of this pathogen. 

Because this study is based on a cohort of 77 suspected case-patients, of whom only 2 laboratory-confirmed cases were identified during the surveillance period, estimates on the basis of identified symptoms are very imprecise (Table 2). However, in the context of an emerging pathogen, reporting such data progressively helps optimize case detection and surveillance systems.

During the 12-month surveillance period, no patients who had respiratory symptoms but no pulmonary parenchymal involvement were positive for MERS-CoV by PCR, and the positive predictive value of signs and symptoms increased with the severity of clinical manifestation. This suggests that the case definitions that were in use during this period (which recommended MERS-CoV testing only for patients who met the epidemiologic criteria and had a severe respiratory illness) were appropriate.

A range of respiratory pathogens were found in those patients that were MERS-CoV negative, highlighting the importance of looking for alternative diagnoses. However, the diagnosis of 1 of the MERS-CoV case-patients was delayed because of an initial diagnosis of influenza. The testing algorithm was subsequently changed to ensure that patients meeting the possible case definition were tested for MERS-CoV if they had an alternative etiology which did not fully explain their clinical manifestation.

The predictive value of the possible case definition depends on the incidence of infection and would be expected to vary across different population groups and change over time, especially in the context of an emerging pathogen. We encourage other countries to similarly report the characteristics of all patients tested for MERS-CoV to improve understanding of the predictive value of different clinical and epidemiologic manifestations in various populations at different times. This will help inform the evolving international public health response to this novel pathogen.
